# The Sociotechnical Ethics of Digital Health: A Critique and Extension of Approaches From Bioethics

**DOI:** 10.3389/fdgth.2021.725088

**Published:** 2021-09-23

**Authors:** James A. Shaw, Joseph Donia

**Affiliations:** ^1^Institute of Health Policy, Management and Evaluation, University of Toronto, Toronto, ON, Canada; ^2^Women's College Hospital, Toronto, ON, Canada

**Keywords:** digital health, bioethics, science and technology studies (STS), telemedicine, ethics

## Abstract

The widespread adoption of digital technologies raises important ethical issues in health care and public health. In our view, understanding these ethical issues demands a perspective that looks beyond the technology itself to include the sociotechnical system in which it is situated. In this sense, a sociotechnical system refers to the broader collection of material devices, interpersonal relationships, organizational policies, corporate contracts, and government regulations that shape the ways in which digital health technologies are adopted and used. Bioethical approaches to the assessment of digital health technologies are typically confined to ethical issues raised by features of the technology itself. We suggest that an ethical perspective confined to functions of the technology is insufficient to assess the broader impact of the adoption of technologies on the care environment and the broader health-related ecosystem of which it is a part. In this paper we review existing approaches to the bioethics of digital health, and draw on concepts from design ethics and science & technology studies (STS) to critique a narrow view of the bioethics of digital health. We then describe the sociotechnical system produced by digital health technologies when adopted in health care environments, and outline the various considerations that demand attention for a comprehensive ethical analysis of digital health technologies in this broad perspective. We conclude by outlining the importance of social justice for ethical analysis from a sociotechnical perspective.

## Introduction

Hope in the promise of digital technologies to contribute to better health and health care continues to grow among many policymakers, health care providers, researchers and technology users around the world (1, 2). Documented perspectives among patients and the public about the use of digital technologies within health care systems are generally positive (3–5), and digital health is viewed at the policy level as a strategy to achieve more efficient and convenient health care delivery (6, 7). The World Health Organization (WHO) established its first global strategy on digital health for the years 2020–2025 (8), and several guidelines have been produced for the evaluation and implementation of digital health technologies in practice (9–11). Despite the persistent challenges in achieving meaningful implementation and use of digital technologies in health care (12), there is a general sense of optimism that digital health will play an important and positive role in promoting health and improving health care into the future (13).

The optimism around the potential of digital health and the commitment to advancing a digital health agenda represent only a partial perspective on the nature and implications of digitally-enabled health care. The COVID-19 pandemic raised awareness of the large body of work documenting the potential role of digital technologies in exacerbating health inequities (14, 15), along with issues such as the influence of large technology companies over public health policy (16). Furthermore, the distribution of digital technologies is linked with important changes to the ways in which people view and structure their lives, and these changes are deeply connected with the practices and institutions of health and health care (3).

These latter observations raise crucial questions about the many consequences of digital health and the ways in which societies might want digital health to develop into the future. These are normative issues connected to imaginaries of the roles that digital health technologies ought to play in promoting health and delivering health care (17, 18). However, research and writing on the normative foundations of digital health and its implications for health and health care has been limited. In this paper, we engage with existing perspectives on digital health from the field of bioethics, and propose an alternative approach to contemplating this important topic. Although there are alternative perspectives in the broad field of applied health ethics on which we could focus in our critique, such as public health ethics (19), we focus specifically on bioethics because it is the dominant approach to ethical analysis for issues in health care and medicine (20). Our critique is thus limited to the body of work analyzing digital health from a conventional bioethical perspective, but the critiques are relevant for related approaches to applied ethics outside of health and medicine as well. Indeed, the boundary around bioethics is porous at best, and many of the approaches addressed in our paper could be viewed as fitting within other fields of applied ethical research in addition to bioethics (e.g., computing ethics).

Digital health refers to a broad collection of technologies and practices that have shifted over time as new technologies have emerged. We align here with Marent and Henwood who bring four forms of technology-enabled care under the definition of digital health (2): telemedicine (synchronous or asynchronous care at a distance), eHealth (searching and exchange of health information), mHealth (use of mobile digital devices for health-related reasons) and algorithmic medicine (incorporating advances in data science and artificial intelligence in health care). In this way, our definition of digital health includes uses of digital technologies for self-tracking or self-care, health information search and exchange, and the direct delivery of health and social care.

## The Bioethics of Digital Health: A Critique

Given the relatively recent emergence of the language of “digital health” as a way to demarcate the broader collection of applications of technologies we place into that category, it is understandable that bioethical analyses of digital health technologies have begun to develop only recently (21). However, several publications exist that have sought to advance scholarship and practice on the bioethics of digital health, as the recent growth of interest has generated a community of scholars proposing various approaches to understanding this domain of bioethical inquiry (22–25). As researchers working in the area of digital health and innovation ethics, we follow this literature closely. In this section, we group this literature into three categories of scholarly contribution, describe each category, and then provide an overarching critique of this literature.

The first type of contribution to the bioethics of digital health literature that we identify we refer to as “**applying ethical theory**.” In this body of literature, scholars adopt the perspective of an existing ethical theory and assess a subset of normatively relevant issues in digital health from that perspective (21, 26). The most common is some form of principlist approach, one that relies on a series of bioethical principles to guide assessment of the ethical implications of any given area of human activity (23, 24, 27, 28). The field of bioethics is dominated by a principlist approach to ethical thinking (20, 29), and it is therefore not surprising that the bioethics of digital health would also be dominated by such an approach. In this approach, contributors tend not to use elaborate justifications for a particular orientation to ethical theory, but rather focus primarily on applying the theory to substantive issues in digital health. For example, in a paper on the ethics of digital phenotyping for health-related uses, Mulvenna et al. simply state that “the four ethical pillars of medicine are autonomy (right to choice), beneficence (doing good), non-maleficence (do no harm), and justice (equal access), and these pillars should not be overlooked when democratizing digital phenotyping” (p. 8) (28). The authors then proceed to focus specifically on the principle of autonomy as the primary focus in their ethical analysis. Contributions in the “applying ethical theory” category have illuminated various dimensions of a set of well-defined normative issues in digital health from the perspectives of commonly known bioethical theories. These normative issues most prominently include privacy, security, data governance, and the distribution of benefits and burdens arising from the use of digital health technologies (16, 26, 30).

The second type of contribution that we identify we refer to as “**translating ethics for practice**.” This type of contribution is focused on enhancing the ability of stakeholders in the digital health ecosystem to understand and apply bioethical concepts in meaningful ways. Translating bioethics for practice is not about analyzing ethical issues from a particular ethical perspective, but rather is about linking ethically-informed statements or principles with actual practices of developing or implementing digital health technologies. For example, Milosevic gave a detailed account of deontologic ethical theory and outlined how specific deontic concepts can be linked directly to the software design process (22). Other contributions aim to further simplify the principlist approach to bioethics and specify its links to various aspects of digital health technology design (27). Approaches in this category aim to simplify and specify the implications of bioethical theory for the actual work of building and deploying digital health technologies (27).

The final type of contribution that we identify we refer to as “**identifying ethical harms**.” Contributions in this category aim to identify and describe the ethically relevant harms or normative issues presented by the domain of digital health. The harms identified in this category of contribution range in their proximity to the technology itself. Harms include issues closer to the technology, such as privacy or trust in digital health technologies (25, 31), and others farther from the technology itself such as the unequal resources available to procure and implement digital health technologies around the world (26). Although the focus of this type of contribution is primarily on the harms, issues or challenges of digital health (32), these are often also linked with the positively stated concepts that can address harms. For example, Vayena et al. specify that where trust is a challenge with digital health technologies, accountability is a strategy to promote trust in the field of digital health over the longer term. Contributions in this category have reinforced the high profile of normatively relevant issues associated with digital health, such as privacy and security, and also encouraged deeper thinking about previously unaddressed issues (26).

The three approaches summarized here have each made important contributions to the global discussion on the ethical challenges presented by digital health technologies and potential strategies to address them. Specifically, they have illuminated the significance of privacy, autonomy, security, consent, transparency, accountability, and fairness, and have explored various approaches to digital health governance. However, they are subject to important critiques that inform our own approach to understanding digital health ethics, based on the critique of bioethics as a field of research and practice (20, 33–35). Our critique relies on two central observations about bioethics as a field of applied ethics for health and medicine that apply directly to our review of literature on digital health ethics. First, that the field of bioethics is built on a foundational belief about the existence of moral universals that are essentially free from the influence of social, cultural, and political realities in different jurisdictions around the world (33, 36, 37). And second, that the common practice in bioethics is to accept the boundaries around a given advancement in health or medical technology that are established by the clinical or technological stakeholders supporting its implementation (34, 35). We address each of these critiques in relation to the digital health ethics literature just summarized.

The first two categories of the bioethics of digital health contributions, “applying ethical theory” and “translating ethics for practice,” rely on a collection of existing ethical theories to address various issues in the field of digital health. These contributions rarely if ever include a detailed justification of the particular ethical approach taken in the analysis, and nor could they; an applied ethics paper is fundamentally not about the philosophical or theoretical justification of a particular ethical theory itself. However, relying on conventional approaches to bioethical theory is increasingly understood as problematic. Critiques of bioethics from the social sciences have clearly illustrated the problems with an assumed universal morality, which as Fox and Swazey have made clear, “is reinforced by the field's commitment to identifying and fostering universal ethical principles that constitute a “common morality” (sometimes referred to as “the common morality”), described by philosophers Tom Beauchamp and James Childress as “the set of norms that all morally serious persons… in all places… share.” (p. 278) (38). Such an orientation toward ethics neglects the fundamental operations of power and culture in shaping moral beliefs (39, 40), and ignores the ways in which bioethics is infused with assumptions that reinforce efforts to maintain the status quo of existing systems of power (37, 41, 42). In our work, we aim to acknowledge these influences on bioethical discourse and promote a self-critical analysis of the assumptions made in ethics work and the particular normatively relevant positions we seek to advance.

The final category of the bioethics of digital health, “identifying ethical harms,” is subject to a related but distinct critique: that bioethics practitioners tend to accept the boundaries placed around ethical discourse by proponents of a given a technology (34). This critique has been advanced clearly by Hedgecoe, who studied the work of bioethicists in the field of pharmacogenetics (34). He identified that bioethicists largely accept the claims made by scientists about the appropriate role of pharmacogenetic advances in medical care, stating, “It is quite clear that bioethicists can be skeptical of these scientific claims. It is just that they are not. Nor is it clear why bioethicists seem content to allow their discourse to remain within its current parameters, and are so unwilling to think in novel ways about the ethical issues raised by pharmacogenetics.” (p. 15) (34).

Work in the bioethics of digital health appears to largely fall victim to the same critique. A series of common issues are frequently identified and discussed from an ethical perspective in relation to digital health technologies, such as those summarized earlier, without questioning the issues presented by such technologies outside of these commonly understood ethical harms. Challenges such as privacy and security can be cast as technical challenges, and it is in the interest of technology developers and other supporters of digital health to keep attention focused on technical challenges that can be contained and addressed using technical approaches (34). Although we acknowledge this is an over-simplification of privacy and security as normative issues, the contrast with issues such as digitally-driven inequities and corporate capture in public health care systems illustrate the immense complexity of potential normative harms that tend to be obscured or avoided in bioethical debate. In our work, we aim to situate the issues most commonly acknowledged in bioethical literature on digital health within the broader context of the social, cultural, and political realities that position them as such in the first place. In order to accomplish the latter goal, we turn to literature in Science and Technology Studies (STS).

## Toward a Sociotechnical Ethics of Digital Health

The sociotechnical approach to the ethics of digital health we propose in our paper arises directly from work in STS. STS is an interdisciplinary field of research that examines the interconnectedness and co-constitution of technology, science and society (43). In this way, STS highlights the people, practices, institutions, and other material realities that shape human understandings of science and technology and their implications for human life (43–45). The term “sociotechnical” refers to the observation that issues pertaining to technologies such as applications of digital health are never solely about the material technology itself, but about the mutual dependencies between technologies and the social arrangements in which they are built and used (46, 47). By the same token, “social arrangements” are always infused with various technologies, ranging from the chairs and whiteboards in design rooms to the smartphone applications and videoconferencing software that mediate human interactions. The term “sociotechnical” thus denotes a broadening of focus from the issues defined by a technology itself, to the broader universe of issues opened up by the recognition that technologies are built and embedded in the social world in ways that profoundly shape and are shaped by human life (48, 49).

We take the phrase sociotechnical system from the work of Selbst et al. on “fairness and abstraction in sociotechnical systems” (47). In their analysis, Selbst et al. outline how a series of biases arise in applications of data-intensive technologies not as a result of the technologies themselves, but as a result of the social and material systems in which they are built and embedded. In addition to their work, the theoretical precursors to our use of the notion of a sociotechnical system are many, but for the purposes of this analysis, we can specify two: infrastructure studies and the political economy of digital data.

Infrastructure studies refers to an approach in the field of STS that examines the often neglected material foundations that make everyday life possible (50, 51). One consequence of focusing on infrastructure is to uncover the inter-connectedness of the infrastructures on which many activities rely by tracing their extension and distance from a given site of analysis. In this way, by looking at digital technologies, we are encouraged to understanding the connected infrastructures on which their uses in health care depend.

In a related vein, work on the political economy of digital data has outlined the typically hidden incentives that characterize the collection, manipulation, and use of data for digital health technologies. In her introduction to a special issue on the topic, Prainsack outlined how studies of the political economy of digital data encourage attention to the institutions that govern and enable particular actors to generate value from data (52). Such an approach urges attention to the workings of power in a globalized capitalist economy that makes demands of local institutions to go along with the most recent capitalist trends. In this way, we also attend to these broader flows of power that shape the field of digital health, and encourage attention to them as important normative issues.

The broadening of perspective from the technology to the sociotechnical system raises attention to potential ethical issues that might have been overlooked from a technology-focused perspective. The sociotechnical approach has the effect of introducing a new series of potential ethical harms that require consideration in ethical analyses of technologies, and in so doing has a higher order impact on our ethical analysis: It more explicitly orients our ethical attention to the question of what kind of world we hope to bring about through the design and deployment of a given technology. As opposed to simply assessing a range of issues that have been determined at the outset to be ethically relevant, this approach allows one to pursue a range of issues more distally connected to the technology that might also require ethical attention; indeed, ethical analysis is considered incomplete until this broader range of ethical issues is acknowledged, particularly in relation to their consequences for the effort to achieve the sort of world we hope to bring about. For example, in beginning with the design of a digital health technology, one might end up analyzing the policy framework in a given jurisdiction related to the presence of for-profit technology corporations influencing health policy (53). The health policy question in this scenario might have important implications for the structure of the health system as a public good, and therefore play an important role in the overarching ethical analysis related to the world we hope to achieve.

In the remainder of this section, we outline a general framework of the sociotechnical domains in which ethical harms of digital health might arise. In the following section, we outline an approach to ethical analysis that contemplates these harms to determine an ethical way forward. We present a typology of *domains* in which ethical harms can be considered in an ethical analysis of digital health technologies from a sociotechnical perspective. The purpose of this framework is simply to provide structure to a sociotechnical ethics approach to digital health, wherein the analyst can develop a sense of where one might look to identify the broader range of ethical issues we have referred to. These domains are not intended to be comprehensive of every feasible area of ethical relevance, but are intended to represent many of the most ethically salient considerations in the ethical analysis of sociotechnical systems in digital health. The domains are summarized in Table 1.

**Table 1:** Domains of analysis in a sociotechnical approach to digital health ethics.

**Domain of analysis**	**Brief description**	**Example ethical issue**
Application Software	The lines of code that constitute a given digital health technology and the health-related practices they compel and discourage	Algorithms that perform with lesser degrees of accuracy for structurally marginalized communities.
Material devices and supply chains	The actual material used to build and distribute the devices through which humans interact with digital health technologies	The negative environmental effects of mining for materials to build digital devices.
Infrastructures	The infrastructure that is required for digital health to function, including material realities such as the buildings in which health care providers work when delivering virtual care, the cables and wires that enable digital signals to travel over distance, and the corporate structures of the organizations that make digital communication available	Lack of access to the Internet for people living in rural and remote areas.
Individual health-related practices	The activities and routines that are compelled by the use of digital health technologies	Adverse mental health implications of continual health-related surveillance.
Interpersonal relationships	Digital health technologies have the capacity to impact interpersonal relationships, through shaping the sources of information, expectations, and modes of interaction available to people	Negative impacts on interpersonal relationships as a result of health-related misinformation shared on social media.
Organizational policies	The structure, function and routines that characterize organizations, and the ways in which these are formalized in organizational rules and policies	The institution of corporate surveillance on staff.
Government policy and regulatory capture	The rules and approaches to governance put in place by state actors, and the influence of digital health stakeholders such as large technology corporations over health-related government policy	The growth in influence of large corporate technology companies over health-related policy.

### Application Software

The lines of code that constitute a given digital health technology and the health-related practices they compel and discourage are certainly of great ethical import. Much work has been done on the topic of the ethics of health-related artificial intelligence (AI) applications, related to the algorithms that determine the functioning of a particular digital health technology (30, 32). Although considerations such as transparency and fairness in the algorithms themselves are certainly important, it is also crucial to acknowledge that the ethical salience of these issues is closely linked with the broader systems of which they are a part (47). Ethical issues at the level of application software include effectiveness, usability, inclusiveness, transparency, and other issues related to the functioning and direct use of the digital health offering (30, 54).

### Material Devices and Supply Chains

The actual material used to build and distribute the devices through which humans interact with digital health technologies are often ignored in ethical analyses, but are highly relevant for a comprehensive perspective on the ethics of digital health. The materials that are used to make smartphones and other digital devices are extracted from the earth and shipped internationally, having the effect of reinforcing low wage labor in low-income countries to benefit mostly large corporations in high-income countries (55). The systems created by such supply chains and the ever-advancing cycle of digital consumption in high-income countries deepens the entrenchment of geopolitical relations, structural racism, and the climate crisis (55), reinforcing their ethical relevance in a broad perspective on the ethics of digital health. Although acknowledging the relevance of the supply chain and the material that makes up the devices required for digital health creates immense challenges for an ethics of digital health, this does not mean they should be excluded from ethical analysis.

### Infrastructures

Digital health relies on hardware and software, as addressed in the first two domains just outlined. However, digital health also relies on a series of different kinds of infrastructure. These infrastructures include the buildings in which health care providers work when delivering virtual care, the cables and wires that enable digital signals to travel over distance, and the corporate structures of the organizations that make digital communication available (56, 57). These and other infrastructures can have crucial ethical implications for digital health, where for example, a lack of high-speed internet availability precludes a particular community from accessing digital health care (54).

### Individual Health-Related Practices

Digital technologies are used in a variety of health-related applications, many of which are intended to promote healthy activity and the management of disease among individual people (3). Digital health technologies are often infused with self-tracking mechanisms that have the impact of encouraging people to self-police their own actions and habits, meaning that they have heightened awareness about whether and how their action align with expected social norms (17, 18). Although the consequences of such self-policing can include enhanced health and prevented illness, there are broader questions to be posed regarding the power of self-tracking and “nudge” technologies to shape and constrain human behavior (58). The power of technology to influence mental well-being as a result of reduced self-esteem, and its power to influence individual actions, are ethically relevant, and should be acknowledged in related ethical analyses of digital health.

### Interpersonal Relationships

Digital health technologies have the capacity to impact interpersonal relationships in variety of ways. One example is the very salient influence of social media applications on public understanding of health-related science and policy (59, 60). Health-related uses of social media have the potential to build interpersonal networks that reinforce particular epistemic viewpoints on health-related issues, with potential damaging effects on public health. A different example is the influence of technology-mediated communication on the relationship between health care provider and patient (61). Although the exact implications of digital health and provider-patient relationships is as yet unclear, the comparison of in-person and digitally-mediated care remains ethically relevant to digital health.

### Organizational Policies

Digital technologies have the potential to dramatically reshape everyday work practices, and therefore to also reshape the structure and function of organizations (62). The ways in which health-related organizations navigate the transition from analog to digital work environments is likely to have substantial implications for the nature of health care work and the nature of patient care (12, 53). The ways in which health care systems operate is very much in the public interest, broadening the range of ethical issues deemed relevant to the ethical analysis of digital health. One important point worth mentioning here is the impact of organizations such as insurance companies that use digital health technologies to collect information about individual behaviors and shape their product offerings accordingly (63). Such practices are made newly effective by advances in digital health technologies, and the role they ought to play in the insurance industry going forward is an organizational policy issue requiring close ethical attention.

### Government Policy and Regulatory Capture

In the context of the growing corporate investment in collecting and analyzing large amounts of health-related data, government regulations become extremely important. More recent advances in data protection law that address health-related data such as the General Data Protection Regulation (GDPR) in the European Union represent important steps toward more comprehensive public protections. However, the rapid advancement of digital health technologies and the corporate practices of the organizations developing them pose important problems even for the GDPR. For example, Marelli et al. outline a series of practices in digital health that are not effectively addressed by the GDPR, including the growing influence of new corporate actors, creating stronger links between health care and lifestyle, increasing reliance on predictive analytics, and social sorting to place technology users into distinct groups (64). Beyond the capacity of existing policy to cover current corporate digital health practices is the growing influence of such corporate actors over the strategy and operations of health care systems. The increasing movement of for-profit technology corporations into the digital health field highlights the urgent need for ethical attention to the conflicting motivations of technology companies and health care systems (16, 65).

These domains in which ethically salient harms might be identified in a sociotechnical approach to digital health ethics represent a departure from the more limited perspective conventionally associated with the field of bioethics (34, 35). A sociotechnical approach encourages the ethical analyst to engage in the work necessary to develop a clearer understanding of the ethical issues presented by a given application of digital health in these various domains. Such an effort might require a review of social science literature on these topics, or new empirical research to uncover the implications of a particular technology and the ways in which it is produced and distributed. However, after the potential harms of such a technology are identified and understood, what would be the approach to adjudicating between those harms and the purported benefits to arriving at a meaningful ethical conclusion? We turn to this important point next.

## Toward the World We Want

If the first important move of a sociotechnical approach to the ethics of digital health is to broaden the scope of issues under consideration, then the second important move is to focus on world-building. As opposed to a phenomenological notion of world-building, by world-building we mean the distributed contributions to producing a particular sort of world that are made by the practices and institutions that enable the sustained development of a given technology. This understanding of world-building is aligned with literature in STS more broadly that attends to the multiple sites of activity that constitute innovation, and the avenues of inquiry from critical political economy approaches that explore whether a particular innovation contributes to the sort of world we hope to bring about (52, 66). The broader focus encouraged by a sociotechnical approach raises awareness of the many ways in which the building and dissemination of a technology can impact the world in ethically relevant ways. In our view, the act of attending to such a broad range of issues invites a summary understanding of the kind of world that is being brought about by the consequences of a technology and the ways in which it is built. In this way, the summative assessment of a technology from the perspective of a sociotechnical ethics relies on an understanding of the sort of world it helps to create, who benefits in that world, and who is disadvantaged. Such an approach prioritizes social justice.

Broadening one's ethical perspective to the many elements of a sociotechnical system has the effect of broadening ones understanding of its normative implications. At this broad level of ethical analysis, we suggest that ethical attention is most naturally focused on world building and the value commitments that support a socially just world for all. When tracing the links in the sociotechnical system, the interconnections between communities that are otherwise considered unconnected come into view, and the interdependence between them becomes ethically salient. Aligned with recent approaches to public health ethics, such an approach calls for ethical attention to the global balance of benefits and burdens in the nested geographies of the local, the national, and the planetary (19). It is this attention to social justice, motivated by commitment to solidarity with the many inter-connected communities affected by a given digital health technology, that characterizes a sociotechnical ethics of digital health. Acting on such an approach requires methods that are familiar to ethically-informed governance in domains separate from but allied to bioethics, and we now turn to concepts from anticipatory governance to describe two of these methods.

### Engagement and Foresight for Sociotechnical Ethics

A sociotechnical approach to the ethics of digital health resonates strongly with the notion of anticipatory governance (67). Guston defines anticipatory governance as, “a broad-based capacity extended through society that can act on a variety of inputs to manage emerging knowledge-based technologies while such management is still possible” (p. 219) (67). This vision conceptualizes anticipatory governance as a distributed practice to be institutionalized in the innovation function of a given society. Although a sociotechnical approach to the ethics of digital health has a more modest aim of informing more immediate ethical analyses, it does draw two linked and important insights from anticipatory governance: the respective importance of “engagement” and “foresight.”

The first insight drawn from anticipatory governance is the importance of engaging diverse lay publics to provide input into the meaning and desirability of a technology (67, 68). Such exchange of ideas and assumptions allows ethicists to better understand (a) the moral assumptions, and (b) knowledge and beliefs held by different publics as they relate to a given technology. Engagement in this way is intended to ensure that under-represented views in innovation, policy, and technology are brought to the discussion and have bearing on the ways in which ethical issues are framed. But engagement is not without its challenges. Defining which publics are to be engaged and securing the resources to do so in a meaningful way require committed action and a supportive, well-resourced context.

The second and related insight from anticipatory governance is the importance of foresight, which has been described in detail in relation to emerging technologies (69–71). In formal literature on anticipatory governance, foresight is incorporated in its full sense as a multi-method practice of identifying current trends and imagining the likelihood and significance of various potential futures (68). These potential futures, identified through consultation with relevant publics, then inform approaches to current governance decisions. In relation to a sociotechnical approach to the ethics of digital health, this is simply about anticipating the potential impacts of the broader range of ethical issues identified in the various sociotechnical domains outlined. The purpose is to anchor decision-making in a clearer understanding of the kind of world that is encouraged by the digital health technology of focus, and what its consequences will be for the inter-connected communities affected by its development and distribution.

The approach articulated here relies on both the engagement of diverse perspectives and the articulation of a future that is more desirable than the present. Neither of these activities can ever be perfect, and thus ethical analyses will always be only partial and incomplete. This is not a “lesser” version of ethical analysis, but from a sociotechnical perspective is simply the only form of ethics that is viewed as possible. It is one that intends to analyze the normative viewpoints of various contributors in relation to the implications of a particular technology, and then to assess their implications for the future. By focusing on social justice for the communities implicated in the development and distribution of a digital health technology throughout the sociotechnical system, the approach aims toward building a better world for all.

The practical implications of the approach we articulate here for health system and organizational leaders relate to the two practical insights just outlined. When health systems are intending to adopt new technologies, they can engage in a systematic process of community engagement to establish a process and governance approach that is meaningful and acceptable to diverse publics. This includes, but is not limited to, those who are structurally marginalized. Such an approach enables health systems to identify issues that might not be understood by those who are in paid positions to procure and implement digital health technologies.

Furthermore, health system leaders can implement an approach that explicitly anticipates the potential negative consequences of adopting technologies for health system stakeholders. Building on the insights of diverse community engagement, such potential negative consequences extend beyond the implications for clinicians who use the technology to the altered roles of administrative staff, leaders, patients, and other stakeholders who contribute to building, distributing, and managing the technology.

Finally, health system leaders can seek out input specifically from ethicists familiar with the unique ethical issues presented by digital health technologies. Although this is an emerging space, specific consultation on the ethics of digital health technologies will become increasingly important as digital health plays a more prominent role in health systems around the world. The broader view we articulate in this paper, and the practical implications we introduce here, help to promote the sustainable and ethical adoption of digital health technologies into the future.

## Conclusion

The sociotechnical ethics of digital health we propose in this paper is based on a critique of the epistemic and normative foundations of much work done on digital health from within the field of bioethics. Informed by such a critique, we propose a view that draws attention to a much wider range of issues represented by the sociotechnical system implicated by a given digital health technology and the well-being of the many communities connected to it. When normative concern is directed to the well-being of these many communities, the value of solidarity and a commitment to social justice become more prominent in ethical analysis. The focus becomes on building a world that is better for all, as opposed to one that is only better for a few privileged stakeholders.

The view we outline in this paper carries forward a position that it is not only the technology itself that requires ethical attention, but also the world into which it is implemented and that it, in turn, creates. As Selbst et al. suggest, “fairness and justice are properties of social and legal systems like employment and criminal justice, not properties of the technical tools within.” (Selbst p. 59). The digital health ethics community will need to engage with this basic insight in determining the most appropriate strategies for the ethical analysis of emerging technologies in health care and public health.

## Data Availability Statement

The original contributions presented in the study are included in the article/supplementary material, further inquiries can be directed to the corresponding author.

## Author Contributions

JS led the drafting of the manuscript. JS and JD developed the ideas. JD provided critical comment and revision to drafts. Both authors approved the final manuscript.

## Conflict of Interest

The authors declare that the research was conducted in the absence of any commercial or financial relationships that could be construed as a potential conflict of interest.

## Publisher's Note

All claims expressed in this article are solely those of the authors and do not necessarily represent those of their affiliated organizations, or those of the publisher, the editors and the reviewers. Any product that may be evaluated in this article, or claim that may be made by its manufacturer, is not guaranteed or endorsed by the publisher.
